# Structural basis for endoperoxide-forming oxygenases

**DOI:** 10.3762/bjoc.18.71

**Published:** 2022-06-21

**Authors:** Takahiro Mori, Ikuro Abe

**Affiliations:** 1 Graduate School of Pharmaceutical Sciences, The University of Tokyo, 7-3-1 Hongo, Bunkyo-ku, Tokyo 113-0033, Japan; 2 Collaborative Research Institute for Innovative Microbiology, The University of Tokyo, Yayoi 1-1-1, Bunkyo-ku, Tokyo 113-8657, Japan; 3 PRESTO, Japan Science and Technology Agency (JST), Kawaguchi, Saitama 332-0012, Japan

**Keywords:** biosynthesis, endoperoxide, enzyme, natural products, X-ray crystallography

## Abstract

Endoperoxide natural products are widely distributed in nature and exhibit various biological activities. Due to their chemical features, endoperoxide and endoperoxide-derived secondary metabolites have attracted keen attention in the field of natural products and organic synthesis. In this review, we summarize the structural analyses, mechanistic investigations, and proposed reaction mechanisms of endoperoxide-forming oxygenases, including cyclooxygenase, fumitremorgin B endoperoxidase (FtmOx1), and the asnovolin A endoperoxygenase NvfI.

## Introduction

**Figure 1:**
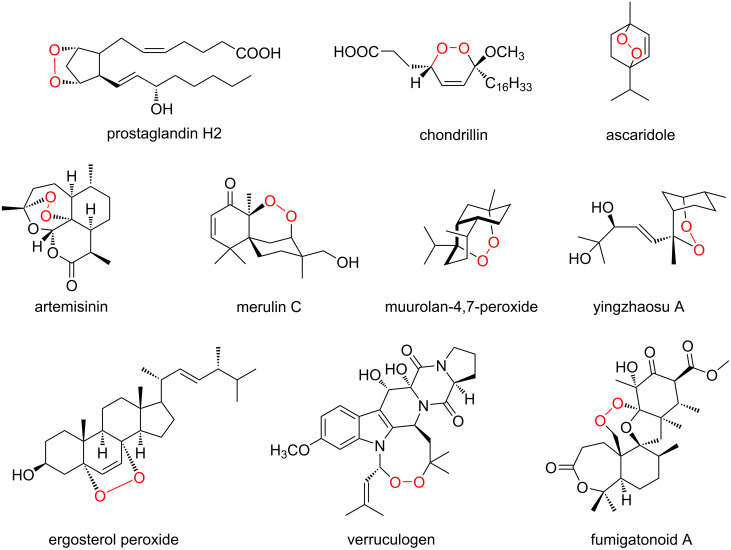
Examples of endoperoxide-containing natural products.

Endoperoxide-containing compounds form a large group of natural products with cyclic peroxide structures [1–5]. These compounds are widely distributed in nature, and many endoperoxide containing alkaloids, terpenoids, and polyketides have been isolated from plants, animals, bacteria, fungi, and other organisms (Figure 1) [6–7]. Because of the high reactivity of the cyclic peroxide O–O bond, these compounds exhibit various biological activities [1–5]. For example, natural and (semi)synthetic endoperoxides with wide structural diversity show antimalarial activity against *Plasmodium falciparum* malaria. In this case, the reductive activation of the endoperoxide ring with the homolytic cleavage of the O–O bond leads to the generation of carbon-centered free radicals that play important roles in antimalarial activity by damaging membranes, inhibiting nucleic acid and protein syntheses, and so on [8–9].

The best studied endoperoxide-containing compound is probably prostaglandin H2 (PGH2), the common precursor of biologically active prostanoids [10–12]. Artemisinin, the antimalarial agent isolated from the plant *Artemisia annua* [8,13–14], and ergosterol peroxides with anticancer and antiviral activities, identified in many fungi, algae, lichens, and plants, also belong to this group [15–17]. Due to the significant biological activities of the endoperoxide-containing natural products, numerous synthetic analyses and biosynthesis of endoperoxide compounds have been reported [18–21]. In some cases; e.g., in the biosynthesis of artemisinin and ergosterol peroxides, a reactive oxygen species (ROS) such as singlet oxygen, which is generated by photosensitizers or visible light, non-enzymatically reacts with the biosynthetic intermediates to produce endoperoxide structures [16,22–23]. However, over the past three decades, only a few endoperoxide-forming enzymes have been identified, including the cyclooxygenases in the biosynthesis of prostaglandins [24], iodide peroxidase in the biosynthesis of ascaridole [25], fumitremorgin B endoperoxidase (FtmOx1) in the biosynthesis of verruculogen [26], and asnovolin A endoperoxygenase NvfI in the biosynthesis of novofumigatonin [27–28]. Among them, although a soluble iodide peroxidase has been isolated from *Chenopodium ambrosioides*, the sequence, structural, and mechanistic analyses have not been vigorously pursued [24].

While many review articles on the structure determination, biological analysis, and synthesis of endoperoxide-containing natural products have been reported [1–7,11,16], the details of the complex biosynthetic enzymes producing these compounds have remained enigmatic. Therefore, this review will focus on the enzymatic synthesis of endoperoxide natural products, by summarizing the recent structural and mechanistic analyses of endoperoxide formation reactions by cyclooxygenases, FtmOx1, and NvfI.

## Review

### COX: Heme-dependent cyclooxygenases in the biosynthesis of prostaglandins

#### Enzyme reaction of COXs

**Scheme 1:**
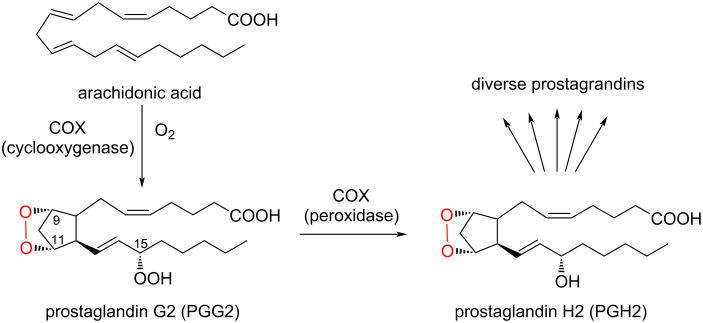
Reactions of COXs.

The cyclooxygenases are the best studied and understood oxygenases among the mammalian oxygenases [29–30]. Mammals have two cyclooxygenase isoforms, COX-1 and COX-2 [31–32], which share ≈60% amino acid identity [33]. Both isoforms catalyze the incorporation of two oxygen atoms into arachidonic acid (AA) to form an endoperoxide between C9 and C11 and a peroxide at C15 to generate prostaglandin G2 (PGG2) (Scheme 1) [24,34]. Subsequently, the 15-hydroperoxide in PGG2 is reduced to produce PGH2. PGH2 is metabolized by downstream enzymes to yield a series of prostaglandins, which play important roles in inflammatory responses [35–37]. Although the active site architectures of COX-1 and COX-2 are not completely identical, the reaction mechanisms and catalytic residues are well conserved. While COX-1 is a constitutive enzyme present in most tissues, COX-2 is an isoenzyme induced in response to tumor promoters, growth factors, and cytokines [38–40]. Therefore, many COX-2 selective inhibitors are clinically used for treatments of inflammation, cancers, and pain [41–43].

#### Crystal structures of COXs

**Figure 2:**
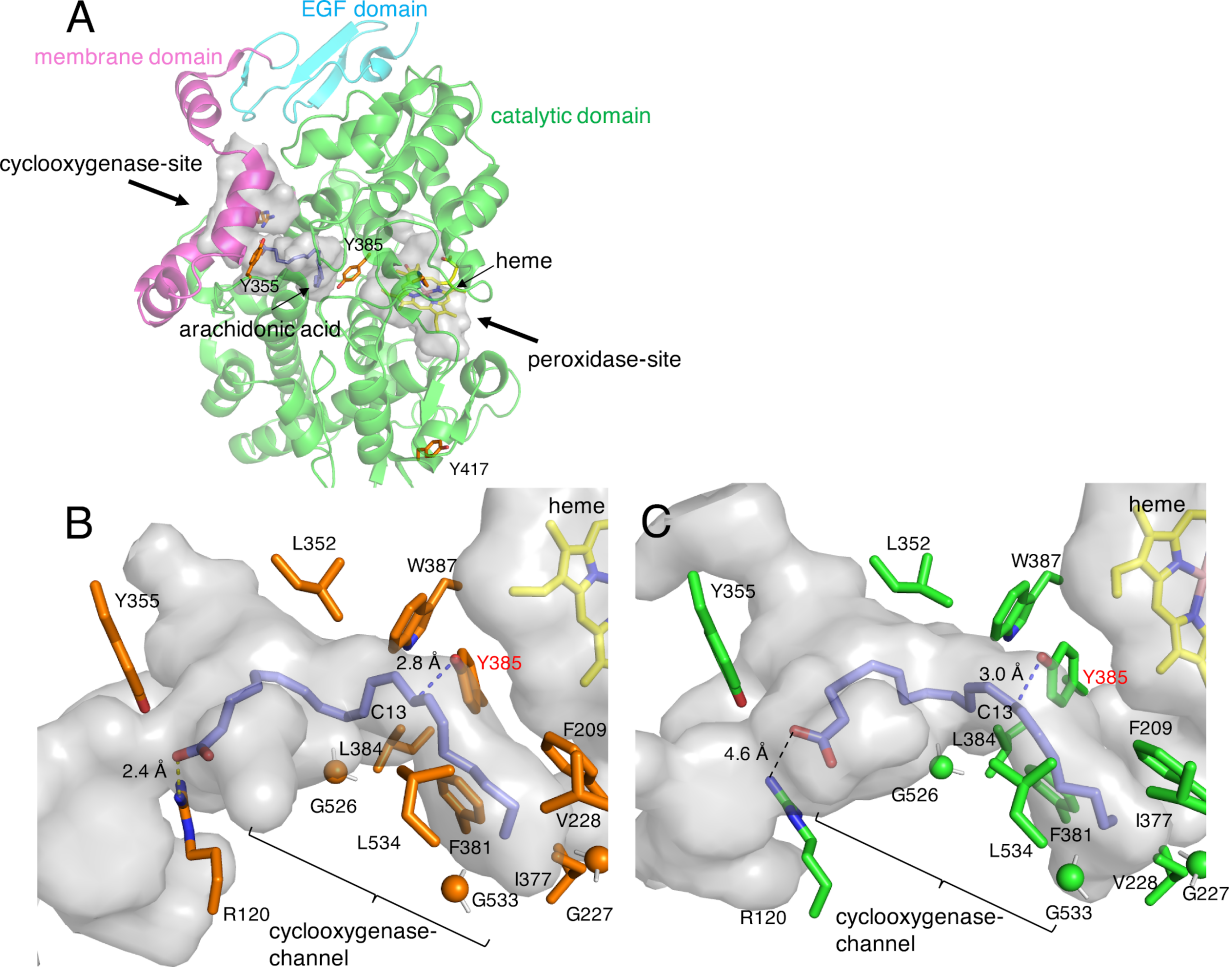
Structures of COXs [52–53]. (A) The overall structure of ovine COX-1. (B and C) Comparison of the cyclooxygenase sites of (B) COX-1 and (C) COX-2 in complex with AA. Yellow dashed lines show hydrogen bond interactions. The blue dashed line shows the distance between Tyr385 and C13 of AA.

The structural basis for the di-peroxide formation reaction by COXs has been substantially elucidated by electron paramagnetic resonance (EPR), kinetic analysis, X-ray crystallography, and mutagenesis experiments [24,44–45]. The structural analyses of mammalian COXs revealed that COX-1 and COX-2 are both glycosylated by post-translational modifications [46–51]. These enzymes form homodimers, and the overall structures of COX-1 and COX-2 superimpose well, with root mean square values of ≈0.9 Å. Each COX monomer contains three domains, including the epidermal growth factor (EGF) domain, the membrane binding domain, and the catalytic domain (Figure 2A) [46–51]. The catalytic domain possesses two active sites, the cyclooxygenase- and heme-dependent peroxidase-sites, which are physically separated. The peroxidase-site activates the catalytic tyrosine residue, while the cyclooxygenase-site catalyzes the formation of di-peroxides. The active site of the peroxidase-site contains a heme cofactor in the solvent-exposed cleft on the opposite side of the membrane binding domain. Although the heme cofactor is located in the peroxidase-site and the active site of peroxidase-site and cyclooxygenase-site are separated, the heme cofactor plays a critical role in both of peroxidase reaction and cyclooxygenase reaction. The active site of the cyclooxygenase-site consists of a deep, L-shaped hydrophobic cavity, referred to as the cyclooxygenase channel, and the entrance of its active site is located on the opposite side from that of the peroxidase-site (Figure 2A).

In the complex structures of COXs with AA (PDB ID: 1CVU, 1DIY, and 3HS5), the carboxylate of the AA is located near the entrance of the cyclooxygenase channel, and the tail of the fatty acyl chain is bound deeply into the narrow hydrophobic channel (Figure 2B and 2C) [48,52–53]. Structural and biochemical studies of ovine COX-1 indicated that the ionic interaction between the carboxylate of AA and Arg120 is required for the reaction (Figure 2B) [54–56]. In contrast, this interaction is not essential in COX-2, suggesting that the hydrophobic interactions between the acyl chain and the active site residues in the cyclooxygenase channel are important for AA binding to COX-2 (Figure 2C) [57–58]. This is one of the major structural differences between COX-1 and COX-2.

#### Catalytic residue in the cyclooxygenase reaction

The formation of a tyrosyl radical during the catalytic cycle was proved by EPR and kinetic analyses [59–61]. Moreover, chemical and molecular biology analyses and a mutagenesis experiment identified the position of the tyrosyl radical. Treatment of the enzymes with tetranitromethane, a reagent for tyrosyl residue nitration, eliminated the cyclooxygenase activity, while the activity was not abolished in the presence of the cyclooxygenase inhibitor indomethacin, indicating that the tyrosine residue(s) is the catalytic center for endoperoxide formation. The sequence analysis of the tetranitromethane-treated enzyme indicated that three tyrosine residues, Tyr355, Tyr385, and Tyr417, were only nitrated in the absence of indomethacin (Figure 2A) [62]. A subsequent mutagenesis study of these residues indicated that the Tyr385 residue plays the catalytic role in the cyclooxygenase reaction. In the crystal structure, the catalytic Tyr385 residue is located at the interface between the active regions of the peroxidase-site and cyclooxygenase-site (Figure 2) [46–51]. Furthermore, the C13 of AA is located near the catalytic residue Tyr385 in the crystal structure, indicating that a tyrosyl radical abstracts the pro-*S* hydrogen atom from C13 of AA (Figure 2B and 2C) [48,52–53].

#### Mechanism of the cyclooxygenase reaction

**Scheme 2:**
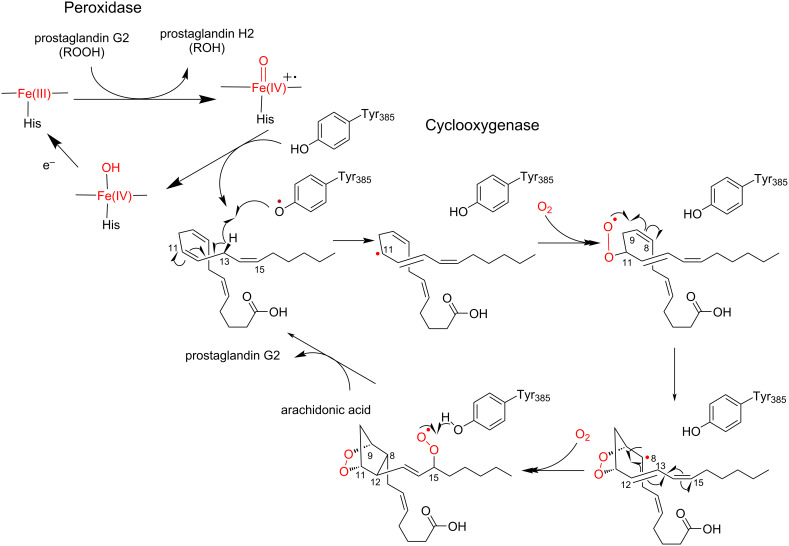
Proposed reaction mechanisms of COXs [24].

The enzyme reaction is initiated upon Tyr385 activation by the oxyferryl heme cation radical, which is generated through the two-electron reduction of PGG2, to form a tyrosyl radical in the active site of the cyclooxygenase-site (Scheme 2) [24,34,63]. The tyrosyl radical then abstracts a C13-pro-*S* hydrogen atom from AA to produce the arachidonoyl radical, which is delocalized over C11 to C15. An oxygen molecule reacts with the C11 radical intermediate to produce a C11-peroxyl radical. The subsequent 5-exo cyclization with a double bond at C8–C9 forms the C8 radical intermediate with the endoperoxide bridge between C9 and C11. The cyclization between C8 and C12 forms a bicyclic radical intermediate, in which the spin density is distributed over C13–C15. The allyl radical at C15 reacts with a second molecular oxygen to afford the C15 peroxyl radical. Finally, the transfer of a hydrogen atom from the catalytic Tyr385 residue quenches the C15 peroxyl radical to yield PGG2 and a tyrosyl radical for the next round of the enzyme reaction. The released PGG2 is accepted by the peroxidase active site, and the 15-hydroperoxyl radical of PGG2 is reduced to generate PGH2.

### FtmOx1: Nonheme iron-dependent endoperoxygenase in the biosynthesis of verruculogen

#### Enzyme reaction of FtmOx1

Fumitremorgin B endoperoxidase (FtmOx1) from *Aspergillus fumigatus* is the first identified nonheme iron and 2-oxoglutarate (Fe/2OG)-dependent endoperoxidase that catalyzes the formation of an endoperoxide in the biosynthesis of verruculogen [26].

**Scheme 3:**
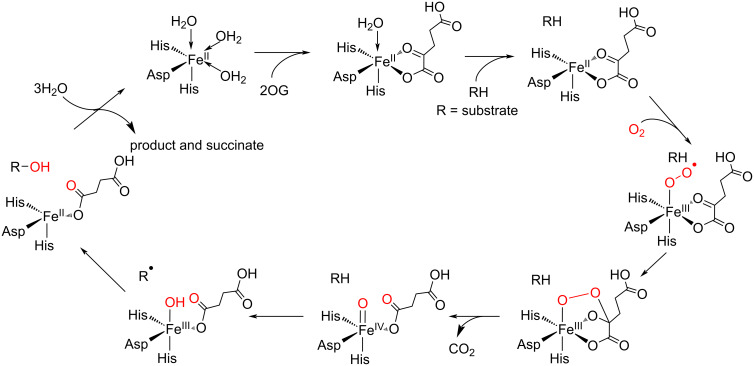
General reaction mechanism of Fe/2OG oxygenases.

Fe/2OG oxygenases utilize Fe(II) as a cofactor and 2OG and O_2_ as co-substrates (Scheme 3) [64–67]. The Fe(II) is coordinated by the conserved 2-His-1-Asp residues and a 2OG in the active site. The binding of a substrate in the active site, where it is close to the Fe(II) center, provides a coordination site for O_2_. Subsequently, the oxidative decarboxylation of 2OG generates a highly reactive Fe(IV)=O species and a succinate byproduct. This Fe(IV)=O abstracts a hydrogen atom from an aliphatic C–H bond of the substrate to generate a radical intermediate. When the enzyme catalyzes the hydroxylation reaction, the radical reacts with the Fe(III)-OH species to form a hydroxylated product.

**Scheme 4:**
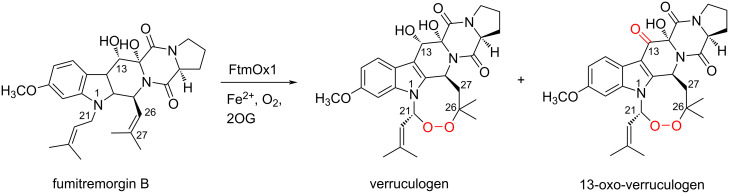
Reaction of FtmOx1 [68–71].

The functional analysis of FtmOx1 indicated that the enzyme accepts fumitremorgin B as a substrate and catalyzes the formation of verruculogen and a C13-oxo product, through the installation of an endoperoxide bridge between C21 and C27 of fumitremorgin B and oxidation of the C13-hydroxy group of verruculogen, respectively (Scheme 4) [68–71]. The single-turnover enzyme reaction of FtmOx1 in the absence of reductants (e.g., ascorbate) indicated that FtmOx1 consumes two oxygen molecules to generate these products in the catalytic cycle. Thus, an O_2_ molecule directly reacts with the radical intermediate of fumitremorgin B, and is incorporated without cleavage of the O–O bond in the enzyme reaction catalyzed by FtmOx1.

Biochemical and biophysical analyses of FtmOx1 have been reported by many different groups, and the structures of FtmOx1 wild type and variants have been solved in the apo and complex forms with 2OG or with 2OG and fumitremorgin B (PDB IDs: 4Y5T, 4Y5S, 6OXH, 6OXJ, 7DE0, 7ETL, and 7ETK) [68–71]. Based on these structural and functional analyses, the reaction mechanism of FtmOx1, and especially the role of the active site tyrosine residues in the catalysis, are heatedly debated.

#### COX-like mechanism of FtmOx1

**Figure 3:**
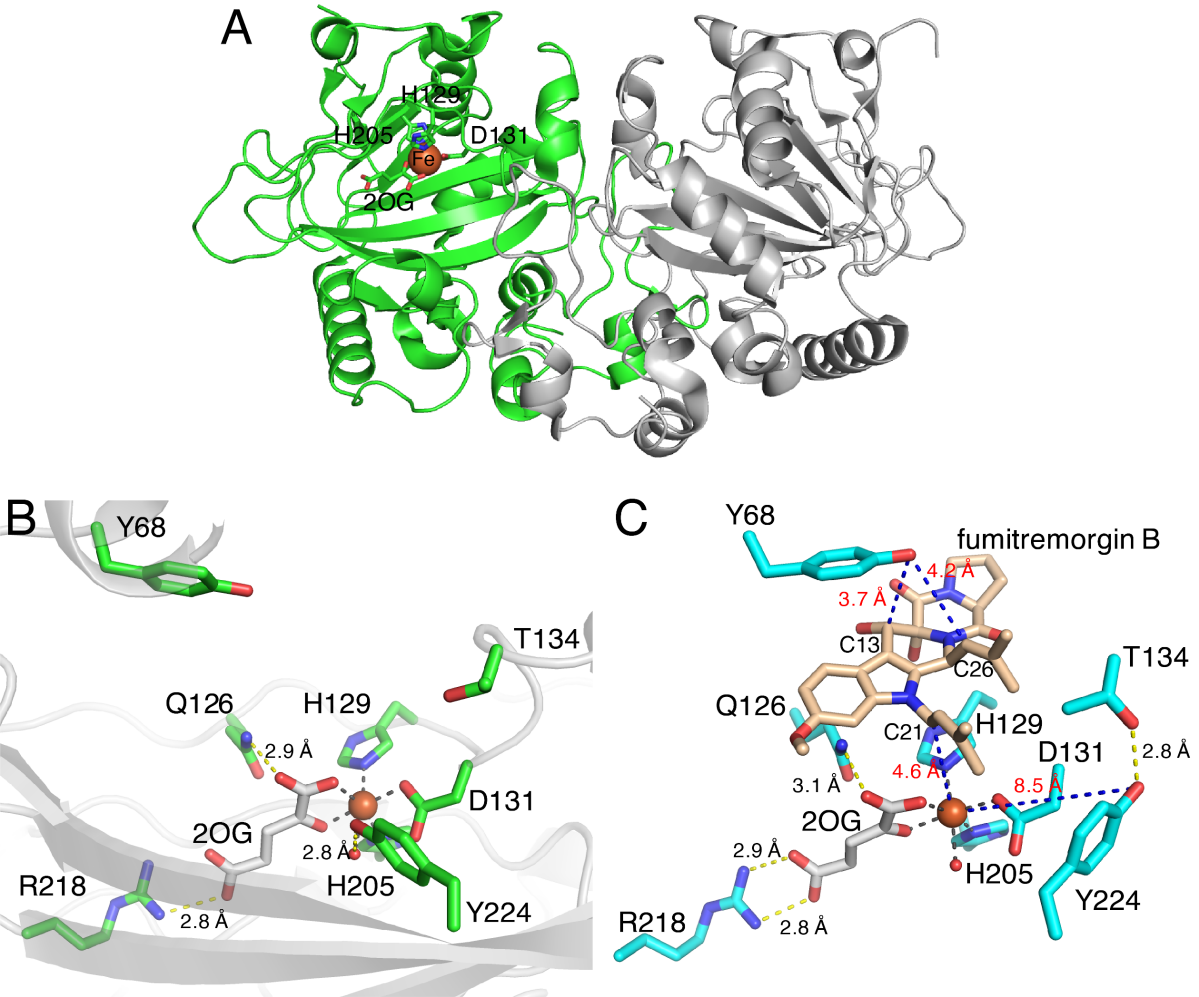
Structure of FtmOx1 [71]. (A) The FtmOx1 binary structure in complex with 2OG. (B and C) Comparison of the active site architectures between (B) the binary structure and (C) the ternary structure. Blue dashed lines show the distances between atoms and yellow dashed lines represent hydrogen bond interactions. Grey dashed lines show the coordination of the iron atom. Iron atoms are depicted by orange spheres.

**Scheme 5:**
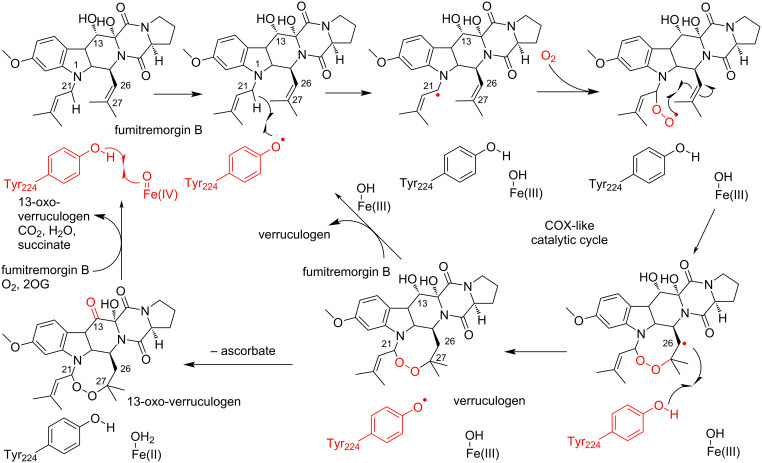
Proposed COX-like mechanism of FtmOx1 [68].

The first crystal structures of FtmOx1 in the apo form and in complex with 2OG were reported in 2015 [68] (this article got retracted [69] in 2021). The overall structure of FtmOx1 exists as a functional homodimer and possesses the double-stranded β-helix (DSBH) fold observed in typical Fe/2OG-dependent oxygenases (Figure 3A). The Fe(II) is coordinated by His129, Asp131, His205, and 2OG in the binary complex structure of FtmOx1 with 2OG. In this structure, Tyr224 is close to the putative oxygen binding site (Figure 3B). Furthermore, the variants in which Tyr224 is substituted with Ala or Phe generates an N-1 dealkylated product, which is non-enzymatically created from C21 hydroxylated products, as a major product. Based on these structural analyses, as well as an EPR analysis of the enzyme reaction of FtmOx1 and a transient ultraviolet–visible (UV–vis) absorption analysis, the group proposed that Tyr224 is involved in the catalytic mechanism of the FtmOx1-catalyzed endoperoxide formation reaction as an intermediary of hydrogen atom transfer (HAT), similar to Tyr385 in the COX reaction. In this COX-like reaction mechanism (Scheme 5), the Fe(IV)=O species oxidizes Tyr224 to form a tyrosyl radical, which abstracts a hydrogen atom from C21 of fumitremorgin B to generate a radical intermediate. The insertion of molecular oxygen at C21 produces a C21 peroxyl radical intermediate, which then reacts with the C26–C27 double bond on another prenyl group to generate the endoperoxide with a C26 radical intermediate. Finally, the radical is quenched by hydrogen atom donation from Tyr224, to form verruculogen and a tyrosyl radical for the next round of the reaction. The tyrosyl radical at Tyr224 is also involved in the oxidation of the C13-hydroxy group of verruculogen to a C13-keto product, in the absence of reductants. Thus, the catalytic Tyr224 in this mechanism plays a similar role to Tyr385 in the COX enzyme reaction. In the paper, the authors also reported the complex structure of FtmOx1 with fumitremorgin B (PDB ID: 4ZON, removed recently). However, re-examination of the electron density map indicated that the density is not fit for fumitremorgin B. Since only biochemical data do not conclusively support the mechanistic role of Tyr224 in catalysis, one of the authors has agreed with the retraction, whereas the other authors stand by their data.

Recent docking and molecular dynamics analyses, as well as DFT calculations of the FtmOx1 reaction, suggested a modified mechanism [72–74]. In this mechanism, the tyrosyl radical at Tyr224 is generated by the Fe(IV)=O species, which abstracts a hydrogen atom at C21 to form an endoperoxide ring through the reaction with an O_2_ molecule, as in the case of the COX-like mechanism. However, the radical quenching at C26 is achieved by reductants such as ascorbate, but not by Tyr224, in the final step of the reaction.

#### CarC-like mechanism of FtmOx1

In 2019, Bollinger and co-workers reported a detailed mechanistic study, including kinetic, UV–vis absorption, and EPR analyses of FtmOx1 wild type and its Y68F, Y74F, Y140F, and Y224F variants [70]. Their results revealed that the Y68F variant did not accumulate the initial tyrosyl radical and the formation of verruculogen was significantly decreased, while the Y224F variant showed similar reaction kinetics and verruculogen productivity to those of wild type FtmOx1. Interestingly, the Y68F variant generated an unidentified product, which was recently determined to be 26-hydroxyverruculogen [75].

**Scheme 6:**
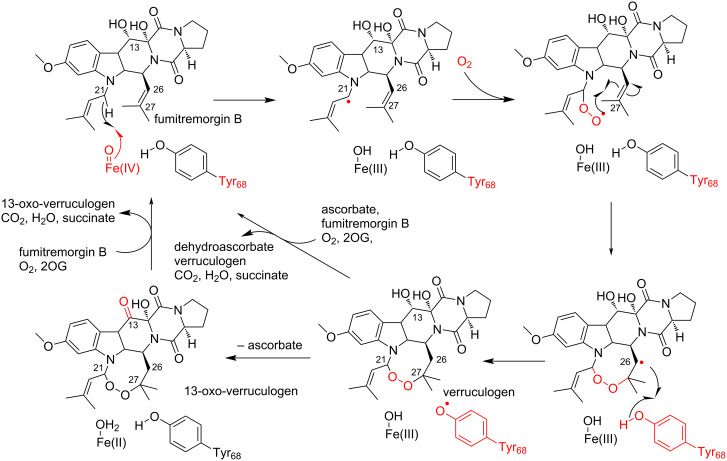
Proposed CarC-like mechanism of FtmOx1 [70].

Based on these observations, they proposed an alternative reaction mechanism. In this proposal, a tyrosyl radical is generated on Tyr68, instead of Tyr224, and this tyrosyl radical donates a hydrogen to the C26 radical intermediate, which is reminiscent of the carbapenem synthase (CarC) reaction mechanism. In this CarC-like mechanism (Scheme 6), the Fe(IV)=O species, but not the tyrosyl radical, first abstracts a hydrogen atom from C21 to form a substrate radical intermediate. The following reaction with molecular oxygen and the formation of an endoperoxide bridge generate the C26 radical intermediate. Finally, HAT from Tyr68 produces verruculogen and a tyrosyl radical at Tyr68, which is quenched by reductants.

The ternary complex structure of FtmOx1 with 2OG and fumitremorgin B was recently reported by Zhou and co-workers [71]. Fumitremorgin B binds in the active site with a planar conformation, through hydrophobic and hydrophilic interactions (Figure 3C). While Tyr68 is located on the protein surface and solvent-exposed, the distance between C21 of fumitremorgin B and the iron center is 4.6 Å and the hydroxy group of Tyr68 is near C26 of fumitremorgin B (Figure 3C). Moreover, Tyr68 is located close to C13 of fumitremorgin B, at a distance of 3.7 Å. The comparison between the binary and ternary complex structures indicated that the Tyr224 residue rotates by 115° toward the opposite side of the iron center and forms a hydrogen bond with T134. The resulting distance between the Tyr224 hydroxy group and the iron center is 8.5 Å. These observations support the CarC-like mechanism, rather than the COX-like mechanism.

Recently, Bollinger and co-workers also reported the incorporation of non-canonical Tyr analogs, including 3-fluorotyrosine, 2,3-difluorotyrosine, 3,5-difluorotyrosine, 3-chlorotyrosine, and 4-aminophenylalanine, at Tyr68 or Tyr224 to analyze the functions of these tyrosine residues [75]. The transient-kinetic, UV–vis absorption, and EPR analyses of FtmOx1 variants containing non-canonical Tyr analogs also indicated that Tyr68, rather than Tyr224, acts as the catalytic residue in the FtmOx1 reaction, supporting the CarC-like mechanism.

### NvfI: Nonheme iron-dependent endoperoxygenase in the biosynthesis of novofumigatonin

#### Enzyme reaction of NvfI

**Scheme 7:**
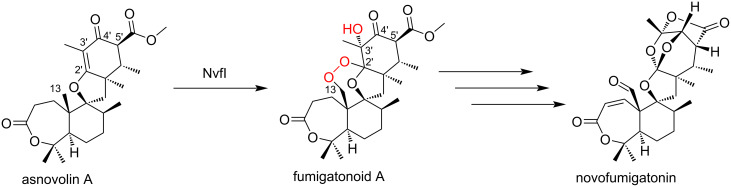
Reaction of NvfI [28].

Asnovolin A endoperoxygenase NvfI is a second example of a 2OG-dependent endoperoxygenase, which is involved in the biosynthesis of novofumigatonin from *Aspergillus novofumigatus* IBT 1680611 [27–28]. The enzyme converts asnovolin A into fumigatonoid A, a biosynthetic intermediate of novofumigatonin, by introducing three oxygen atoms, including a hydroxy group at C3' and an endoperoxide bridge between C13 and C2' (Scheme 7). Although the in vitro assay of NvfI indicated that the enzyme is a 2OG-dependent endoperoxidase, NvfI shares a low amino acid sequence similarity with FtmOx1 (only 17%) and a phylogenetic analysis indicated that it is located in a different clade from FtmOx1.

**Scheme 8:**
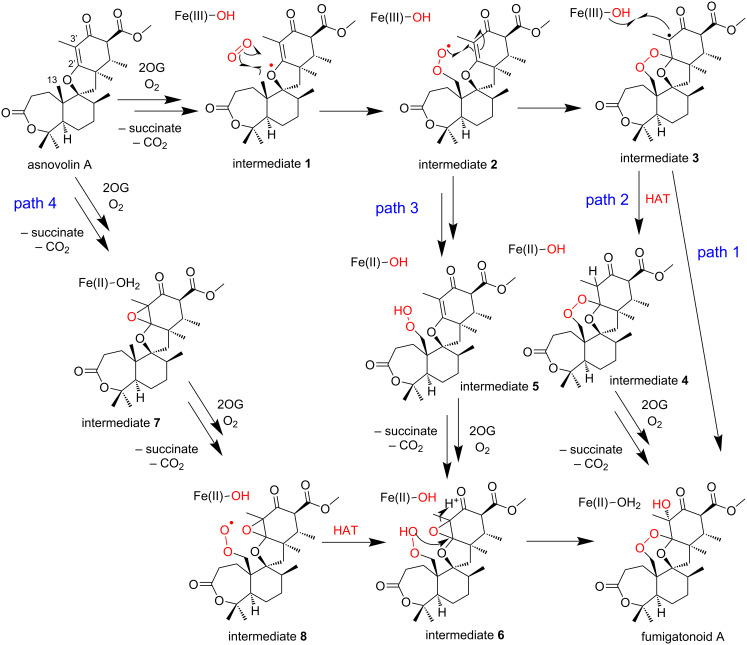
Possible reaction pathways leading to fumigatonoid A [28].

Prior to the structure–function analysis of NvfI, four pathways were proposed for the formation of fumigatonoid A from asnovolin A (Scheme 8, path 1). The Fe(IV)=O species abstracts a hydrogen atom from C13 of asnovolin A to form radical intermediate **1**. Subsequently, it reacts with molecular oxygen to form a peroxyl radical intermediate **2**. The peroxyl radical attacks C2' to generate intermediate **3**, which contains an endoperoxide bridge and a C3' radical. Finally, the hydroxylation at C3' by the Fe(III)-OH species yields fumigatonoid A (path 2). At the stage of intermediate **3** in path 1, HAT from an active site residue or reductant to the C3' radical in intermediate **3** generates intermediate **4**. Then, the hydroxylation at C3' forms fumigatonoid A (path 3). The C13 peroxide intermediate **5** is generated from intermediate **2**, which is subsequently epoxidated at C2'–C3' to form intermediate **6**. The following cyclization reaction from the peroxide generates fumigatonoid A (path 4). The epoxide formation reaction occurs first at C2'–C3' of asnovolin A (intermediate **7**), and then the peroxide formation (intermediate **8**) and cyclization (intermediate **6**) reactions produce fumigatonoid A.

The stoichiometric analysis of 2OG and O_2_ indicated that one equivalent of 2OG and two equivalents of O_2_ are consumed to generate fumigatonoid A, suggesting that the installation of three oxygen atoms onto asnovolin A occurs in a single turnover of the enzyme reaction. Thus, pathways 2–4, which require two equivalents of 2OG for the formation of fumigatonoid A, are unlikely. Further experiments with ^18^O_2_ and H_2_^18^O suggested that three oxygen atoms are enzymatically incorporated into fumigatonoid A, in which the oxygen atoms of the endoperoxide are derived from the O_2_ molecule and the C3' hydroxy group most likely originates from the solvent water. Although the oxygen atom in the hydroxylation reaction is usually from molecular oxygen, the oxygen atom in the Fe(III)-OH species can be exchanged with the solvent water [76]. Therefore, the fact that almost all of the oxygen atoms in the Fe(III)-OH species exchanged with the solvent in the enzyme reaction of NvfI suggested the presence of a long-lived C3' radical and the hydroxy ligand during the reaction.

#### Structure of NvfI

**Figure 4:**
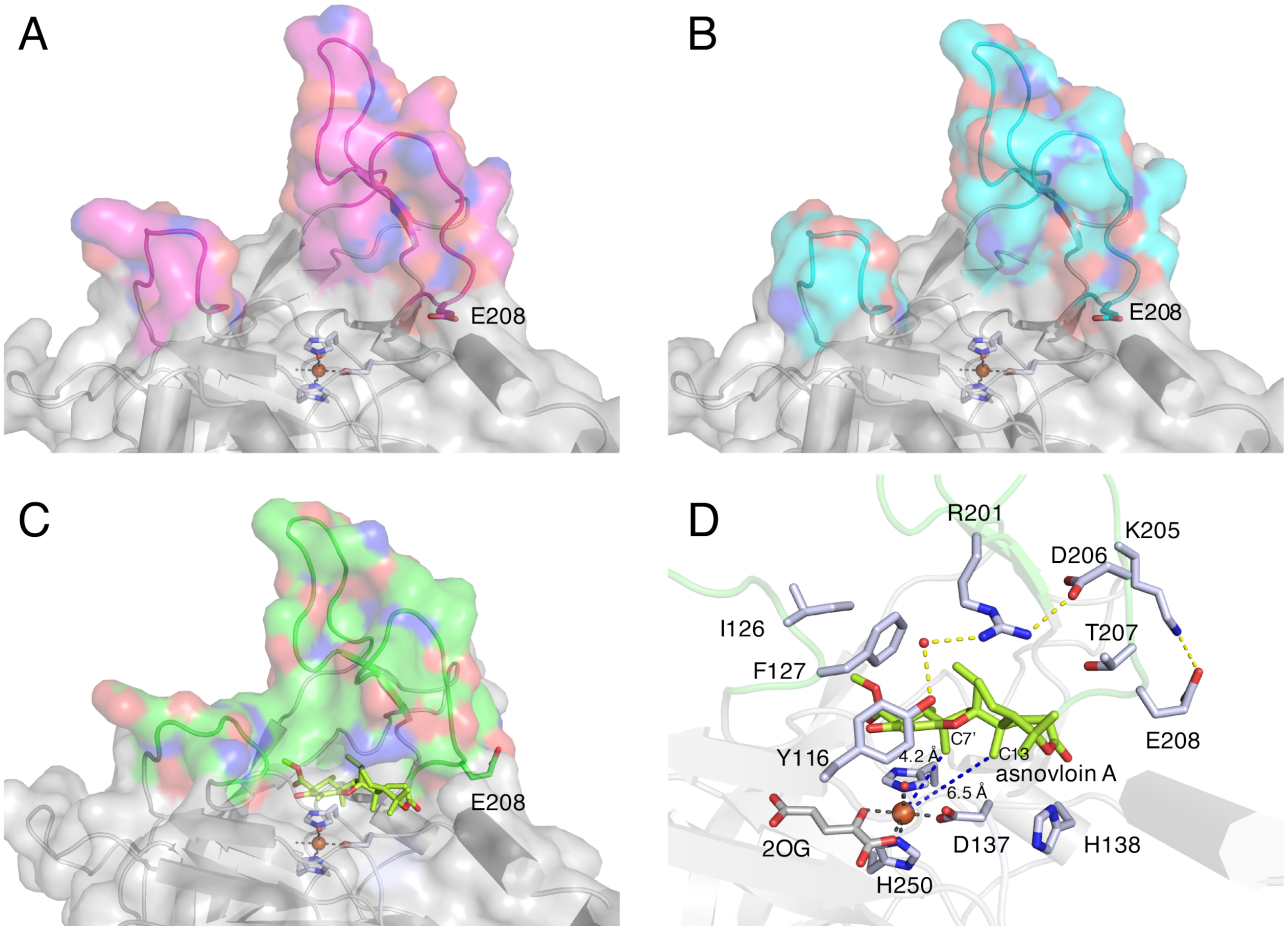
Structure of NvfI [28]. (A–C) Conformational changes of loop regions: (A) open conformation, (B) partially closed conformation, and (C) closed conformation of NvfI. (D) The active site of NvfI in complex with asnovolin A. Blue dashed lines show the distances between atoms and yellow dashed lines represent hydrogen bond interactions. Iron atoms are depicted by orange spheres.

In the structural analysis of NvfI, three different active site conformations (open, partially closed, and closed) were observed (PDB IDs: 7DE2, 7EMZ, and 7ENB) (Figure 4A–C) [28]. The conformations of the loop regions between Ser122-Gly128 and Trp199-Pro209 were altered by soaking with the substrate. The substrate asnovolin A binds in the closed conformation through a hydrogen bond network with active site residues (Figure 4D). In this binding mode, C7' of the substrate is 4.2 Å away from the iron center, which is shorter than the distance between the iron center and C13 (6.5 Å). This spatial arrangement is not reasonable for the formation of fumiganoid A, because the initial step should be the abstraction of a C13 hydrogen atom by the Fe(IV)=O species. Considering the conformational changes of the active site and the docking simulation of the substrate in the active site of the partially closed conformation, the substrate binding mode in the crystal structure apparently shows a different stage of the reaction. The conformational changes of the loops, especially the flipping of Glu208, would contribute to alterations of the binding mode of the substrate and the long-lived radical on the intermediate and Fe(III)-OH species (Figure 4A–C). Interestingly, a mutagenesis experiment of the active site residues of NvfI, including Tyr116, suggested that the enzyme does not employ any active site residues for the HAT step.

#### Reaction mechanism of NvfI

**Scheme 9:**
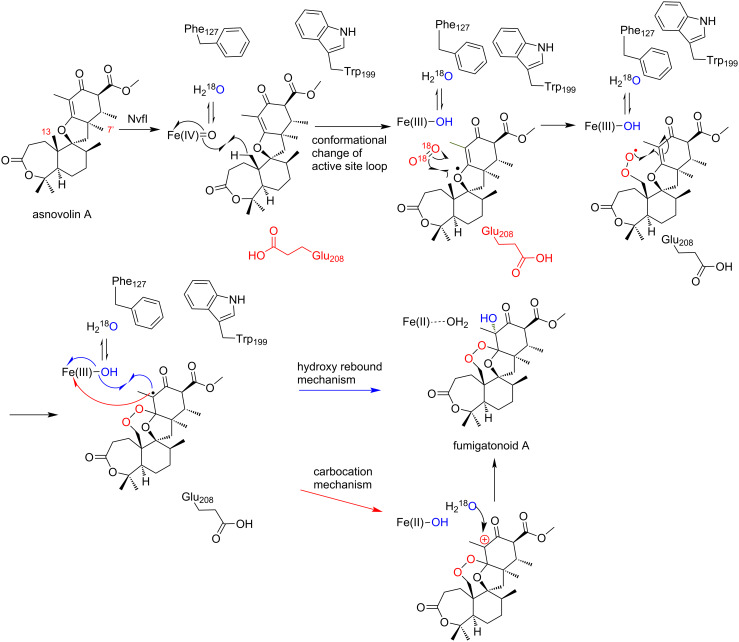
Another possible reaction pathway for the formation of fumigatonoid A [28].

Based on these observations, we propose the mechanism of the NvfI-catalyzed endoperoxide formation reaction (Scheme 9). First, the substrate asnovolin A binds in the active site of the open conformation. The binding of asnovolin A would be a driving force for the conformational change of the loop to form a partially closed conformation. Then, the abstraction of a hydrogen atom from C13 is catalyzed by the Fe(IV)=O species. Here, the active site residue Glu208 would contribute to determining the position of the hydrogen atom abstraction, by forming steric hindrance with the A-ring of the substrate. Subsequently, further conformational changes of the active site residues on flexible loops would relocate the C13 radical intermediate, to prevent the hydroxy-rebound from the Fe(III)-OH species. Alternatively, the C13-radical reacts with molecular oxygen to form a peroxyl radical intermediate, which undergoes radical addition to C2' to generate an intermediate containing an endoperoxide bridge and a C3' radical. Finally, hydroxylation at C3' by the Fe(III)-OH species produces fumigatonoid A. Considering the catalysis by 2OG-dependent oxygenases, the radical mechanism of the hydroxylation is plausible. However, it is also possible that the water addition occurs on the C3' carbocation, which is generated by one electron transfer to the ferric iron from the C3' radical intermediate (carbocation mechanism in Scheme 9). Notably, in this last step, the stereochemistry of the hydroxylation reaction is regulated by the enzyme to be the *R*-configuration. These biochemical and biophysical analyses of NvfI suggested that NvfI catalyzes the endoperoxide formation reaction through a different mechanism from those of COX and FtmOx1. Further computational studies and mechanistical studies are now in progress in our laboratories to clarify the molecular dynamics of the active site during the enzyme reaction.

## Conclusion

Endoperoxide compounds have recently attracted keen attention as a source of new drug leads, due to their unique structures and remarkable biological activities. To date, the total synthesis of endoperoxide-containing natural products and the synthesis of chemical reagents with an endoperoxide bridge have been reported [77–78]. The classical method for endoperoxide synthesis is through cycloadditions of dienes and alkenes, using singlet oxygen. Furthermore, cyclizations of hydroperoxides with pendant alkenes or alkynes have also been reported, by using a metal catalyst or Brønsted-acid catalysis [79–81]. However, the efficient regio- and stereoselective installation of the endoperoxide structure is still challenging, because of the increased reactivity of activated oxygen/peroxides and the high sensitivity of peroxide bridges to reductants. In this aspect, the chemoenzymatic synthesis would be a useful approach to synthesize endoperoxide-containing compounds with high regio- and stereoselectivities. The structural and mechanistic analyses of the enzymes for endoperoxide formation reviewed here would provide useful information about the engineering and design of enzymes for this application.

Although more than 200 endoperoxide-containing natural products have been isolated, only three enzymes responsible for the formation of endoperoxides have been characterized. The structural and mechanistic analyses of endoperoxide-forming enzymes, COX, FtmOx1, and NvfI, indicated that these enzymes employ distinct reaction mechanisms, suggesting that the enzymatic endoperoxide formation reactions individually evolve in a substrate-dependent manner. This fact makes it difficult to identify the endoperoxide-forming enzymes by a sequence-based genome mining approach. While it is possible that many endoperoxide compounds are produced through non-enzymatic oxidation by ROS, future biosynthetic analyses of endoperoxide natural products will lead to the discovery of novel endoperoxide-forming pathways and generate more comprehensive molecular insights into their remarkable chemistries. Finally, the detailed structural and mechanistic investigations of these enzymes will provide an excellent basis for the development of biocatalysts to generate novel compounds with endoperoxides for future drug discovery.
